# The impact of depression on satisfaction with prenatal care in Kazakhstan during COVID-19 pandemic: Raushan Alibekova

**DOI:** 10.1093/eurpub/ckac130.117

**Published:** 2022-10-25

**Authors:** G Kossybakova, R Alibekova, B Crape, A Kuntuganova

**Affiliations:** Department of Medicine, Nazarbayev University School of Medicine, Nur-Sultan, Kazakhstan

## Abstract

**Background:**

Depression and anxiety are common during perinatal periods, representing a considerable public health concern during global pandemic. Only few studies have examined the influence of emotional disturbances on satisfaction with maternity care. This study aimed to assess the prevalence of satisfaction with prenatal care and to examine its association with depression and anxiety among pregnant women in Kazakhstan.

**Methods:**

Participants were recruited to this online cross-sectional survey in the outpatient clinic in Novoshamalgan, Almaty region (n = 174) in December-January, 2022. A single-item measure of satisfaction was used by asking a question “How satisfied are you with health services?”, scored on a 5-point Likert scale. A simplified 2-item Edinburgh Postpartum Depression Scale and 2-item Generalized Anxiety Disorder scale were used to measure depressive and anxiety symptoms.

**Results:**

Majority of women were very satisfied or satisfied (n = 128; 74%), while less than a third of the sample (n = 46; 26%) were dissatisfied with the received care. The prevalence of depression in the total sample was higher, compared to the prevalence of anxiety (34% versus 18%). Multivariable logistic regression showed that dissatisfaction with prenatal care was associated with older age, not attending check-ups regularly, and being employee of private company or student. Depression increased the odds of being dissatisfied by 2.6 times (95% CI 1.19∼5.79); while obstetric issues and anxiety were not associated with satisfaction. Perception of women about inadequate solution of the problem of antenatal depression was a significant predictor of dissatisfaction (AOR 6.87; 95% CI 1.81∼26.12).

**Conclusions:**

Depressed women in our study were less satisfied with prenatal care. Further investigation of the perception of women about specific aspects of perinatal health services is suggested. Providing a quality, patient-centered care is needed to support pregnant women during the current pandemic.

**Key messages:**

- To improve the quality of prenatal care, health providers should address not only physical concerns, but also psychosocial issues and symptoms of depression of pregnant women during current pandemic.
- Although prenatal care is accessible to all pregnant women in Kazakhstan, development of patient-centered perinatal care is needed, focusing on communication skills and quality of interaction.

